# Type 2 diabetes genetic loci informed by multi-trait associations point to disease mechanisms and subtypes: A soft clustering analysis

**DOI:** 10.1371/journal.pmed.1002654

**Published:** 2018-09-21

**Authors:** Miriam S. Udler, Jaegil Kim, Marcin von Grotthuss, Sílvia Bonàs-Guarch, Joanne B. Cole, Joshua Chiou, Christopher D. Anderson on behalf of METASTROKE and the ISGC, Michael Boehnke, Markku Laakso, Gil Atzmon, Benjamin Glaser, Josep M. Mercader, Kyle Gaulton, Jason Flannick, Gad Getz, Jose C. Florez

**Affiliations:** 1 Diabetes Unit, Massachusetts General Hospital, Boston, Massachusetts, United States of America; 2 Center for Genomic Medicine, Massachusetts General Hospital, Boston, Massachusetts, United States of America; 3 Broad Institute of MIT and Harvard, Cambridge, Massachusetts, United States of America; 4 Department of Medicine, Harvard Medical School, Boston, Massachusetts, United States of America; 5 Barcelona Supercomputing Center (BSC), Joint BSC-CRG-IRB Research Program in Computational Biology, Barcelona, Spain; 6 Department of Pediatrics, University of California San Diego, San Diego, California, United States of America; 7 Department of Biostatistics and Center for Statistical Genetics, University of Michigan, Ann Arbor, Michigan, United States of America; 8 Institute of Clinical Medicine, Internal Medicine, University of Eastern Finland and Kuopio University Hospital, Kuopio, Finland; 9 Faculty of Natural Sciences, University of Haifa, Haifa, Israel; 10 Department of Medicine; Albert Einstein College of Medicine, Bronx, New York, United States of America; 11 Department of Genetics, Institute for Aging Research, Albert Einstein College of Medicine, Bronx, New York, United States of America; 12 Endocrinology and Metabolism Service, Hadassah-Hebrew University Medical Center, Jerusalem, Israel; 13 Department of Genetics, Boston Children’s Hospital, Boston, Massachusetts, United States of America; University of Cambridge, UNITED KINGDOM

## Abstract

**Background:**

Type 2 diabetes (T2D) is a heterogeneous disease for which (1) disease-causing pathways are incompletely understood and (2) subclassification may improve patient management. Unlike other biomarkers, germline genetic markers do not change with disease progression or treatment. In this paper, we test whether a germline genetic approach informed by physiology can be used to deconstruct T2D heterogeneity. First, we aimed to categorize genetic loci into groups representing likely disease mechanistic pathways. Second, we asked whether the novel clusters of genetic loci we identified have any broad clinical consequence, as assessed in four separate subsets of individuals with T2D.

**Methods and findings:**

In an effort to identify mechanistic pathways driven by established T2D genetic loci, we applied Bayesian nonnegative matrix factorization (bNMF) clustering to genome-wide association study (GWAS) results for 94 independent T2D genetic variants and 47 diabetes-related traits. We identified five robust clusters of T2D loci and traits, each with distinct tissue-specific enhancer enrichment based on analysis of epigenomic data from 28 cell types. Two clusters contained variant-trait associations indicative of reduced beta cell function, differing from each other by high versus low proinsulin levels. The three other clusters displayed features of insulin resistance: obesity mediated (high body mass index [BMI] and waist circumference [WC]), “lipodystrophy-like” fat distribution (low BMI, adiponectin, and high-density lipoprotein [HDL] cholesterol, and high triglycerides), and disrupted liver lipid metabolism (low triglycerides). Increased cluster genetic risk scores were associated with distinct clinical outcomes, including increased blood pressure, coronary artery disease (CAD), and stroke. We evaluated the potential for clinical impact of these clusters in four studies containing individuals with T2D (Metabolic Syndrome in Men Study [METSIM], *N* = 487; Ashkenazi, *N* = 509; Partners Biobank, *N* = 2,065; UK Biobank [UKBB], *N* = 14,813). Individuals with T2D in the top genetic risk score decile for each cluster reproducibly exhibited the predicted cluster-associated phenotypes, with approximately 30% of all individuals assigned to just one cluster top decile. Limitations of this study include that the genetic variants used in the cluster analysis were restricted to those associated with T2D in populations of European ancestry.

**Conclusion:**

Our approach identifies salient T2D genetically anchored and physiologically informed pathways, and supports the use of genetics to deconstruct T2D heterogeneity. Classification of patients by these genetic pathways may offer a step toward genetically informed T2D patient management.

## Abstract 2

Using a clustering Bayesian approach applied to GWAS, Jose Florez and colleagues identify traits and loci associated with type 2 diabetes that may be used to classify patients.

## Author summary

**Why was this study done?:**

- Clinical experience and physiological phenotyping suggest that type 2 diabetes (T2D) is a heterogeneous disease.
- Recent studies leveraged phenotypic data to identify subtypes of individuals with T2D but have not included genetic data as a driving component of the clustering process.
- Previous efforts to cluster T2D genetic loci used unsupervised hierarchical clustering, a “hard clustering” method that restricts the membership of a given genetic locus to a single cluster.

**What did the researchers do and find?:**

- We applied a novel “soft clustering” method termed Bayesian nonnegative matrix factorization to cluster variant-trait associations ascertained from publicly available genome-wide association studies for 94 known T2D variants and 47 diabetes-related traits.
- We identified five novel robust clusters of T2D loci, two related to insulin deficiency and three related to insulin resistance, and showed that these clusters are differentially enriched for relevant tissue-specific enhancers and promoters.
- In up to 17,365 individuals with T2D from four distinct studies, about 30% of individuals have a genetic risk score in the top decile of uniquely one cluster, and these individuals have cluster-specific phenotypic traits that distinguish them from others with T2D.

**What do these findings mean?:**

- Soft clustering of genetic loci associated with T2D produced biologically supported mechanistic pathways, illuminating how loci might impact T2D risk.
- Classification of individuals by these genetic pathways may offer a step toward genetically anchored but physiologically informed diagnosis, surveillance, and management of patients with T2D.

## Introduction

Type 2 diabetes (T2D) is a complex disease affecting the world’s population at epidemic rates and whose pathophysiology remains incompletely understood. Approximately 30.3 million (9.4%) people in the United States have diabetes, with T2D thought to account for 90%–95% of all diagnoses [1,2]. Despite recognized heterogeneity in patient phenotypes and responses to treatment, T2D management strategies remain largely impersonalized.

In an attempt to deconstruct the heterogeneity of T2D, recent studies have performed cluster analysis of individuals using serum biomarkers and clinical data to identify T2D subgroups [3,4]. These studies offer exciting directions for future research but are also limited by the nature of the variables included in analyses. For example, Ahlqvist and colleagues [4] clustered individuals using six variables measured shortly after diabetes diagnosis, including glycated hemoglobin [HbA1c], glutamate decarboxylase antibodies, and body mass index (BMI); notably, these variables change with disease progression and treatment, and thus application of this clustering approach to clinical practice is of uncertain utility when patients are evaluated at a different time in the disease course or after treatment has been initiated. Additionally, it is not clear whether clinical biomarkers used in cluster analyses to date are causal, consequential, or coincidental in the disease process.

In contrast to other serum biomarkers, germline genetic variants associated with T2D are more likely to point to T2D causal mechanisms and remain constant regardless of developmental stage, disease state, or treatment. Over the past decade, genome-wide association studies (GWAS) and other large-scale genomic studies have identified over 100 loci associated with T2D, causing modest increases in disease risk (odds ratios generally <1.2) [5–9]. These genetic loci offer insight into biological pathways causing T2D, but for most of these loci, the causal variant(s) and the mechanism by which the locus causes T2D remain unknown, limiting opportunities for clinical translation.

As GWAS have been conducted across multiple traits, there exists an opportunity to leverage multi-variant-trait association patterns to elucidate likely shared disease mechanisms, based on the assumption that genetic variants that act along a shared pathway will have similar directional impact on various observed traits. For example, amongst genetic variants impacting insulin resistance, Yaghootkar and colleagues identified a set of 11 variants associated with a particular directional pattern of traits in GWAS. This set of 11 insulin resistance–increasing alleles was felt to represent a “lipodystrophy-like” fat distribution subgroup of insulin resistance variants reminiscent of monogenic lipodystrophy, because they were associated with increased fasting insulin and triglyceride levels but decreased high-density lipoprotein (HDL) cholesterol, adiponectin, and BMI [10].

As with insulin resistance, T2D-associated genetic variants have been assessed using a similar multi-variant-trait clustering approach; however, the resultant clusters have had limited clinical interpretability to date. Three efforts to perform clustering of T2D loci have been published by Dimas and colleagues [11], focusing on glycemic traits, and recently by Scott and colleagues [6] and Mahajan and colleagues [9], both including BMI and lipid traits in addition to glycemic traits. In these analyses, unsupervised hierarchical clustering was performed on T2D variants using their associations with respective traits. While these approaches generated some biologically suggestive clusters of genetic loci, determining the number and boundaries of clusters using unsupervised hierarchical clustering remains rather subjective. Additionally, in these analyses, many variants could not be clustered (more than half of all loci included in [6,11]), including loci with known mechanisms, tissue specificity, and physiological impact (e.g., those in *HNF1A* and *CILP2/TM6SF2*). The unsupervised hierarchical clustering model applied in these previous efforts requires that a variant be included in only one pathway, so-called “hard-clustering,” and was limited by the GWAS datasets available for diabetes-related traits. We were interested in investigating a more flexible model that would allow a variant to impact more than one biological pathway and hypothesized that this might improve cluster interpretability, using the most up-to-date GWAS datasets available for metabolic traits.

In this paper, we test whether a germline genetic approach can be used to deconstruct T2D heterogeneity. First, we ask whether genetic variants can be categorized into groups representing likely disease mechanistic pathways. We apply the novel clustering method Bayesian nonnegative matrix factorization (bNMF) to enable a "soft clustering," whereby a variant can be associated to more than one cluster, which has been used previously in cancer genomics [12–15]. To confirm that these groups of variants represent distinct entities with predicted biological relevance, we assess tissue specificity for enhancer or promoter enrichment. Finally, we ask whether the novel clusters of genetic loci we identified are of any clinical consequence, which we assess in four independent cohorts of individuals with T2D.

## Materials and methods

### Variant and trait selection

The study design proceeded as per a prespecified analysis plan, modified to reflect the additional number of genetic variants associated with T2D over the course of the study as well as the increased availability of GWAS datasets for metabolic traits. To obtain a comprehensive set of genetic variants associated with T2D, we started with the set of 88 variants reaching genome-wide significance aggregated by Mohlke and Boehnke [5], and then added 37 additional loci that were reported in subsequent T2D large-scale genetic studies [6,7,9]. At some loci, there have been reports of multiple distinct signals [6,8]; we included 9 additional variants representing distinct signals at 6 loci (*ANKRD55*, *DGKB*, *CDKN2A*, *KCNQ1*, *CCND2*, and *HNF4A***)**. Of the 125 T2D variants considered, we selected a subset of 94 representative variants based on the condition that either the variant or a proxy of the variant had an association with T2D in the DIAGRAM version 3 (DIAGRAMv3) Stage 1 meta-analysis [16] with *P* < 0.05 **(S1 Table)**. Proxies of variants were required to either be in linkage disequilibrium (*r*^*2*^ > 0.6) with an original T2D variant published at genome-wide significance, be the lead SNP at the T2D locus in DIAGRAMv3, or reach genome-wide significance in DIAGRAMv3. Because DIAGRAMv3 contains study populations of mostly European ancestry, focusing on variants with at least nominal significance in this dataset helped ensure that these variants would also be associated with other traits in published GWAS, as most of the GWAS populations were also of predominantly European ancestry. Additionally, variants (other than those representing distinct signals at a locus) were conservatively excluded if they fell within 500 kb of another variant on the list. Given that C/G and A/T alleles are ambiguous and can lead to errors in aligning alleles across GWAS, we avoided inclusion of ambiguous alleles, choosing proxies instead.

For the 94 T2D-associated variants, the T2D risk–increasing alleles were identified and all future analyses used the aligned T2D risk–increasing alleles. Summary association statistics for additional traits whose GWAS meta-analyses were publicly available were aggregated for each variant (**S2 Table**). These traits included glycemic traits available through the Meta-Analyses of Glucose and Insulin-related traits Consortium (MAGIC) (fasting insulin, fasting glucose, fasting insulin adjusted for BMI, 2-hour glucose on oral glucose tolerance test [OGTT] adjusted for BMI [2hrGlu adj BMI], glycated hemoglobin [HbA1c], homeostatic model assessments of beta cell function [HOMA-B] and insulin resistance [HOMA-IR], incremental insulin response at 30 minutes on OGTT [Incr30], insulin secretion at 30 minutes on OGTT [Ins30], fasting proinsulin adjusted for fasting insulin, corrected insulin response [CIR], disposition index [DI], and insulin sensitivity index [ISI]) [17–23]. Anthroprometric traits were publicly available through the Genetic Investigation of ANthrometric Traits (GIANT) consortium (BMI, height, waist circumference [WC] with and without adjustment for BMI, and waist-hip ratio [WHR] with and without adjustment for BMI) [24–26]. Additional birth weight and length GWAS summary statistics were obtained from the Early Growth Genetics (EGG) consortium [27,28]. GWAS results for visceral and subcutaneous adipose tissue were available from VATGen consortium [29], as well as results for percent body fat [30] and heart rate [31]. Finally, serum laboratory values were available for the following traits: lipid levels (HDL cholesterol, low-density lipoprotein [LDL] cholesterol, total cholesterol, triglycerides) [32], leptin with and without BMI adjustment [33], adiponectin adjusted for BMI [34], urate [35], Omega-3 fatty acids [36], Omega-6-fatty acids [37], plasma phospholipid fatty acids in the de novo lipogenesis pathway [38], and very long-chain saturated fatty acids [39]. To reduce noise in the cluster analysis, traits were only included if at least one variant was associated with the trait at a Bonferroni-corrected threshold of significance *P* < 5 × 10^−4^ (0.05/94).

In addition to the above traits used in the clustering process, single-variant association results with ten clinical outcomes were also aggregated (**S2 Table**): ischemic stroke and its subtypes (large vessel disease, small vessel disease, and cardioembolic) [40]; coronary artery disease (CAD) [41]; renal function as defined by estimated glomerular filtration rate (eGFR); urine albumin-creatinine ratio (UACR); chronic kidney disease (CKD) [42]; and systolic blood pressure (SBP) and diastolic blood pressure (DBP) [43].

### bNMF clustering

We generated standardized effect sizes for variant trait associations from GWAS by dividing the estimated regression coefficient beta by the standard error, using the GWAS summary statistic results. To address the marked differences in sample size across studies and allow for a more uniform comparison of phenotypes across studies, we scaled the standardized effect sizes by the square root of the study size, as estimated by mean sample size across all SNPs, forming the variant-trait association matrix **Z** (94 by 47).

To enable an inference for latent overlapping modules or clusters embedded in variant-trait associations, we modified the existing bNMF algorithm to explicitly account for both positive and negative associations. Each column of **Z** split into two separate entities: one contained all positive z-scores, the other all negative z-scores multiplied by −1, and setting zero otherwise, leading to the association matrix **X** (94 by 94) comprised of doubled traits with positive and negative associations to variants. Then, bNMF factorizes **X** into two matrices, **W** (94 by *K*) and **H**^T^ (94 by *K*), with an optimal rank *K*, as **X** ~ **WH**, corresponding to the association matrix of variants and traits to the determined clusters, respectively. This mathematical framework enables bNMF to tackle both positive and negative associations with no loss of information, while keeping its nonnegativity constraint. Determining the proper model order *K* is a key aspect in balancing data fidelity and complexity. Conventional nonnegative matrix factorization (NMF) requires the model order as an input, or it may be determined post–data processing, but bNMF is designed to suggest an optimal *K* best explaining **X** at the balance between an error measure, ||**X**−**WH**||^2^, and a penalty for model complexity derived from a nonnegative half-normal prior for **W** and **H** [12–14]. In addition, bNMF exploits an automatic relevance determination technique to iteratively regress out irrelevant components in explaining the observed data **X**. More specifically, each column in **W** and each row in **H** is strictly tied together by a common relevance weight corresponding to the variance of half-normal priors, which is also sampled from the inverse-gamma hyperpriors. The exact objective function optimized by bNMF is a posterior, which has two opposing contributions from the likelihood (Frobenius norm) and the regularization penalty (*L*_*2*_-norm of **W** and **H** coupled by the relevance weights) [12]. During the search for the maximum posterior solution, bNMF iteratively prunes irrelevant components in explaining **X** through a shrinkage of relevance weights and enables an optimal inference for the number of clusters, *K*, as well as a sparse representation of **W** and **H** at the balance between data fidelity (Frobenius norm or sum of squared error) and complexity (regularization penalty). The defining features of each cluster are determined by the most highly associated traits, which is a natural output of the bNMF approach. bNMF algorithm was performed in R Studio for 1,000 iterations with different initial conditions, and the maximum posterior solution at the most probable K was selected for downstream analysis. The results of clustering provide cluster-specific weights for each variant (**W**) and trait (**H**).

### Trait and outcome associations with each cluster

An association of the genetic risk score (GRS) for each cluster with each GWAS trait or outcome (“GWAS GRS”) was performed using inverse-variance weighted fixed effects meta-analysis using summary statistics from GWAS, as has been done previously [10]. We tested for associations between the GWAS GRSs and traits to confirm clustering results because traits were included in the clustering process; outcomes were not included in the clustering process.

For these analyses, the top set of strongest-weighted variants for each cluster were included in the model using a cutoff weighting of 0.75, which was determined by two independent approaches involving modeling of cluster weights. First, the top-weighted trait in each cluster was assessed with variants in the corresponding cluster in a stepwise approach; a meta-analysis of the variants with the top-weighted trait was performed, starting with all variants and removing sequential variants from lowest to highest weighted until the local minimum meta-analysis *P*-value was obtained. As a second approach, we assessed the distribution of the variant clustering weights for each variant across all clusters with the goal of identifying optimal cutoffs to define the beginning of the “long tail” of cluster weights, representing less informative variants for each cluster **(S3 Fig)**. We plotted the change in consecutive clustering weight deltas sorted in descending order and reasoned that the long tail should start just after the last significant difference in consecutive weights **(S4 Fig)**. Therefore, all clustering weight deltas were ordered in descending order, and the top 5% were considered to be significant deltas. The last significant delta is shown in **S4 Fig** with an arrow, and it corresponds to clustering weight of 0.75.

Ten outcomes were assessed, which were independent of the traits included in the bNMF clustering. A conservative significance threshold was set at 1×10^−3^ using a Bonferroni correction for 10 traits and 5 clusters (0.05/50).

### Functional annotation and enrichment analysis

We calculated Bayes factors for all variants 500 kb upstream and downstream with *r*^*2*^ greater than 0.1, with the index variant (100% credible set) at each locus from effect size estimates and standard errors, using the approach of Wakefield [44]. We then calculated a posterior probability for each variant by dividing the Bayes factor by the sum of all Bayes factors in the credible set. We obtained previously published 13-state ChromHMM [45] chromatin state calls for 28 cell types, excluding cancer cell lines [46]. For each cell type, we extracted chromatin state annotations for enhancer (Active Enhancer 1, Active Enhancer 2, Weak Enhancer, Genic Enhancer) and promoter (Active Promoter) elements.

We assessed enrichment of annotations first within clusters and second across clusters. For within-cluster analysis, we overlapped cell type annotations with credible set variants for loci in each cluster. We then calculated a cell type probability for the cluster as the sum of posterior probabilities of variants in cell type enhancers or promoters divided by the number of loci in the cluster. For the across-cluster analysis, we overlapped cell type annotations with credible set variants for all loci. For each cluster, we then calculated a cell type probability as the sum of posterior probabilities of all variants in cell type enhancers or promoters in the cluster divided by the total number of loci in the cluster.

We derived significance for cell type probabilities for each cluster using a permutation-based test. Within each cluster, we permuted locus and cell type labels and then recalculated cell type probabilities, as above. For across-cluster analysis, we permuted cluster labels for each locus and then recalculated cell type probabilities for the permuted clusters, as above. For the five loci (*ADCY5*, *CDC123*, *HNF4A*, *HSD17B12*, and *CCND2*) represented in multiple clusters, we ensured that each locus was only represented once per cluster. We then used the cell type probabilities derived from 1 million permutations as a background distribution and performed a one-tailed test to ascertain significance for each cell type.

### Study populations

#### Metabolic Syndrome in Men Study

From this cross-sectional study of Finnish men [47], we analyzed data from 487 individuals with T2D previously ascertained for genotyping as part of the T2D-GENES initiative [48]. Genotyping was performed using Illumina HumanExome-12v1_A Beadchip, and imputation was performed using the Haplotype Consortium Reference Panel [49] using the Michigan Imputation Server [50]. Written consent was provided by all study participants.

#### Diabetes Genes in Founder Populations (Ashkenazi) study

We analyzed data from 509 individuals with T2D from this study previously ascertained for genotyping as part of the T2D-GENES initiative [48]. Briefly, the study consists of individuals of Ashkenazi Jewish origin selected from two separate DNA collections: (1) one affected individual selected per family from a genome-wide, affected-sibling-pair linkage study [51]. Families in which both parents were known to have diabetes were excluded. (2) Patients ascertained by the Israel Diabetes Research Group between 2002 and 2004 from 15 diabetes clinics throughout Israel. For this study, only T2D patients with age of diagnosis between 35 and 60 were selected [52]. Genotyping was performed using Illumina Cardio-Metabo Chip, and imputation was performed using the Haplotype Consortium Reference Panel [49] using the Michigan Imputation Server [50]. Written consent was provided by all study participants.

#### The Partners Biobank

The Partners HealthCare Biobank maintains blood and DNA samples from more than 60,000 consented patients seen at Partners HealthCare hospitals, including Massachusetts General Hospital, Brigham and Women's Hospital, McLean Hospital, and Spaulding Rehabilitation Hospital, all in the Boston area of Massachusetts [53]. Patients are recruited in the context of clinical care appointments at more than 40 sites and clinics, and also electronically through the patient portal at Partners HealthCare. Biobank subjects provide consent for the use of their samples and data in broad-based research. The Partners Biobank works closely with the Partners Research Patient Data Registry (RPDR), the Partners' enterprise data repository designed to foster investigator access to a wide variety of phenotypic data on more than 4 million Partners HealthCare patients. Written consent was provided by all study participants. Approval for analysis of Biobank data was obtained by Partners IRB, study 2016P001018.

T2D status was defined based on “curated phenotypes” developed by the Biobank Portal team using both structured and unstructured electronic medical record (EMR) data and clinical, computational, and statistical methods [54]. Cases were selected by this algorithm to have T2D with PPV of 90% and required to be of at least age 35 to further minimize misclassification of T2D diagnosis. Additional phenotypic data were extracted using the most recent value within the past 5 years. Genomic data for 15,061 individuals were generated with the Illumina Multi-Ethnic Genotyping Array. The genotyping data were harmonized and quality controlled with a three-step protocol, including two stages of SNP removal and an intermediate stage of sample exclusion. The exclusion criteria for variants were (i) missing call rate ≥0.05, (ii) significant deviation from Hardy-Weinberg equilibrium (*P* ≤ 10^−6^ for controls and *P* ≤ 10^−20^ for the entire cohort), (iii) significant differences in the proportion of missingness between cases and controls *P* ≤ 10^−6^, and (iv) minor allele frequency <0.001. The exclusion criteria for samples were (i) gender discordance between the reported and genetically predicted sex, (ii) subject relatedness (pairs with ≥0.125, from which we removed the individual with the highest proportion of missingness), (iii) missing call rates per sample ≥0.02, and (iv) population structure showing more than four standard deviations within the distribution of the study population, according to the first four principal components. Phasing was performed with SHAPEIT2 [55] and then imputed with the Haplotype Consortium Reference Panel [49] using the Michigan Imputation Server [50].

#### The UK Biobank

UK Biobank (UKBB) is a prospective cohort of about 500,000 recruited individuals from the general population aged 40–69 years in 2006–2010 from across the United Kingdom, with genotype, phenotype, and linked healthcare record data. Individuals in UKBB underwent genotyping with one of two closely related custom arrays (UK BiLEVE Axiom Array or UK Biobank Axiom Array) consisting of over 800,000 genetic markers scattered across the genome. Additional genotypes were imputed centrally using the Haplotype Reference Consortium (HRC) reference panel [49], 1000G phase 3 [56], and UK10K reference panel [57], as previously reported [58]. SNPs used for computing the GRS were imputed only with the HRC reference panel. We restricted the analysis to a subset of unrelated individuals of white European ancestry, constructed centrally using a combination of self-reported ancestry and genetically confirmed ancestry, by projecting UKBB genetic principal components on to 1000G phase 3 reference principal component space. We focused on individuals with T2D, based on a recently developed algorithm of information at the baseline visit that took into account diabetes diagnosis, diabetes medication use, and age at diagnosis [59]. We expanded upon this definition to include touchscreen self-reported diagnosis and diagnosis and medication information provided at repeat visits, and removed individuals with reported “age of diabetes diagnosis” less than 40 to further reduce possible contamination with type 1 diabetes diagnoses. A total of 14,813 individuals determined by the algorithm to have “probable” or “possible” T2D were included in our analyses. Documented consent was provided by all study participants.

#### Individual-level analyses of individuals with T2D

Individual-level analyses were performed using data from Metabolic Syndrome in Men Study (METSIM), the Diabetes Genes in Founder Populations (Ashkenazi) study, the Partners Biobank, and UKBB. Analyses were restricted to individuals with T2D and Caucasian ancestry.

All SNPs were genotyped or imputed with high quality (*r*^*2*^ values >0.95). SNPs were included in genetic risks scores as allele dosages. GRSs were generated for each cluster by multiplying a variant’s genotype dosage by its cluster weight. Only the top-weighted variants falling above the threshold, as defined above, were included in the GRS for each cluster. Logistic regression and linear regression were performed in Stata v14.2, adjusting for sex, age, and principal components. Results from the multiple cohorts were meta-analyzed using an inverse-variance weighted fixed effect model. Comparison of traits across the five subgroups of individuals with top decile cluster GRSs were compared using the Kruskal-Wallis non-parametric test for continuous traits, except for percentage female sex, which was compared with the chi-squared test.

## Results

### Clustering suggests five dominant pathways driving diabetes risk

**Fig 1:**
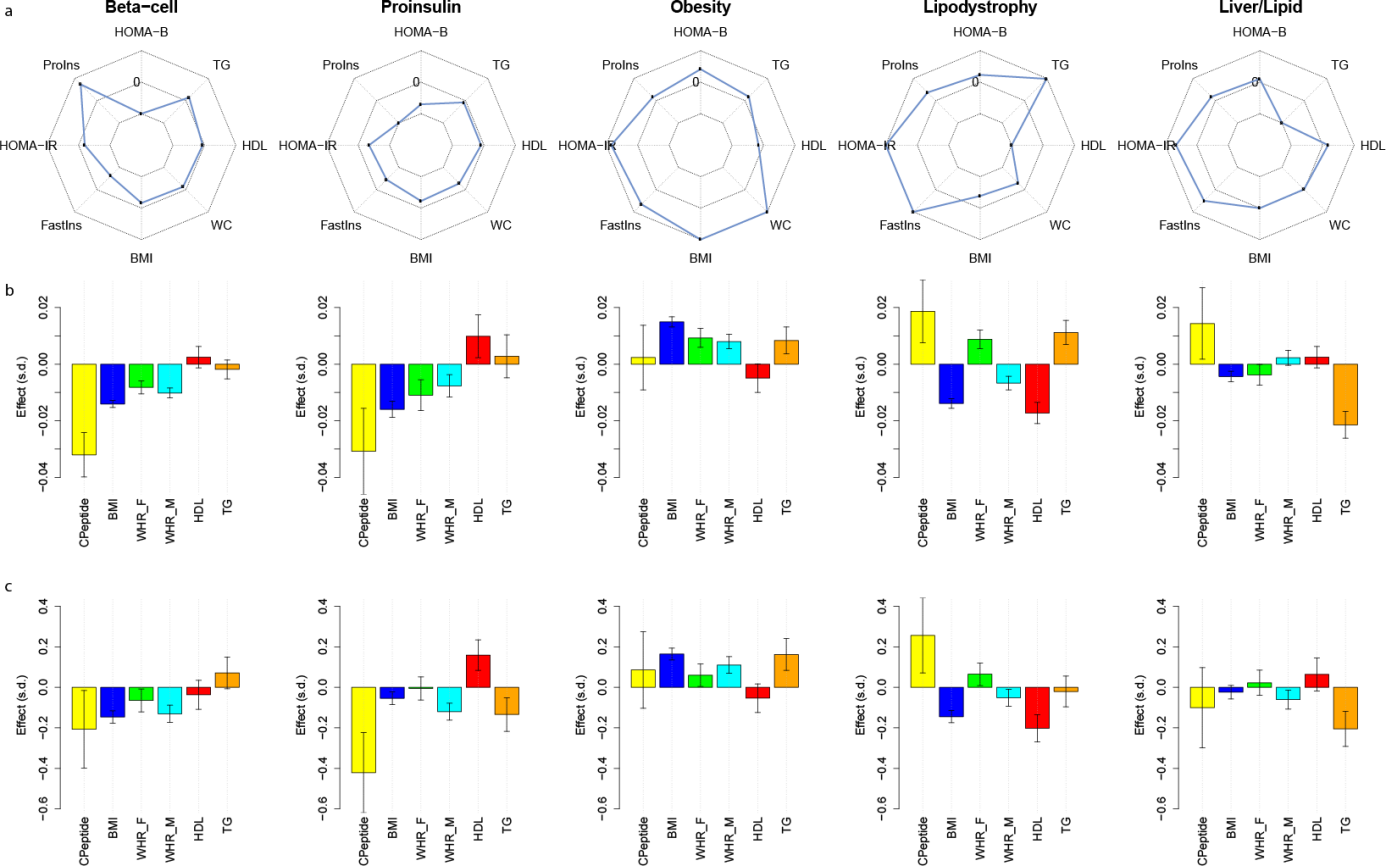
Cluster-defining characteristics. (A) Standardized effect sizes of cluster GRS-trait associations derived from GWAS summary statistics shown in spider plot. The middle of the three concentric octagons is labeled “0,” representing no association between the cluster GRS and trait. A subset of discriminatory traits are displayed. Points falling outside the middle octagon represent positive cluster-trait associations, whereas those inside it represent negative cluster-trait associations. (B) Associations of GRSs in individuals with T2D with various traits. Results are from four studies (METSIM, Ashkenazi, Partners Biobank, and UK Biobank) meta-analyzed together. Effect sizes are scaled by the raw trait standard deviation. (C) Differences in trait effect sizes between individuals with T2D having GRSs in the highest decile of a given cluster versus all other individuals with T2D. Results are from the same four studies meta-analyzed together. Effect sizes are scaled by the raw trait standard deviation. BMI, body mass index; Fastins, fasting insulin; GRS, genetic risk score; GWAS, genome-wide association study; HDL, high-density lipoprotein; HOMA-B, homeostatic model assessment of beta cell function; HOMA-IR, homeostatic model assessment of insulin resistance; METSIM, Metabolic Syndrome in Men Study; ProIns, fasting proinsulin adjusted for fasting insulin; TG, serum triglycerides; T2D, type 2 diabetes; WC, waist circumference; WHR-F, waist-hip ratio in females; WHR-M, waist-hip ratio in males.

Clustering of variant-trait associations was performed for 94 genetic variants and 47 traits derived from GWAS using bNMF clustering, with identification of five robust clusters present on 82.3% of iterations (**S1** and **S2 Figs**; **S1–S4 Tables)**. In 17.3% of the iterations, the data converged to four clusters, in which Cluster 2 was subsumed into Cluster 1. As bNMF clusters both variants and traits, the top-weighted traits can be used to help define the underlying mechanism in each cluster. The five clusters appeared to represent two mechanisms of beta cell dysfunction and three mechanisms of insulin resistance **(Fig 1A, Table 1, S5 Table)**.

**Table 1:** Associations of cluster genetic risk scores and selected GWAS traits.

	Beta Cell *N* Loci = 30		Proinsulin *N* Loci = 7		Obesity *N* Loci = 5		Lipodystrophy *N* Loci = 20		Liver/Lipid *N* Loci = 5	
Trait	beta	*P*-value	beta	*P*-value	beta	*P*-value	beta	*P*-value	beta	*P*-value
Adiponectin	−0.0005	0.55	−0.0019	0.37	−0.0007	0.74	**−0.0114**	**3.34E**^**−23**^	−0.0007	0.77
BMI	−**0.0026**	**6.0×10**^**−5**^	**−0.0080**	**3.1×10**^**−8**^	**0.0396**	**9.7×10**^**−157**^	**−0.0079**	**1.81E**^**−21**^	0.0001	0.94
Bodyfat	−0.0016	0.11	−0.0061	4.5×10^−3^	**0.0247**	**2.1×10**^**−25**^	**−0.0120**	**9.04E**^**−22**^	−0.0031	0.26
CIR	−**0.0584**	**7.1×10**^**−43**^	−0.0234	0.014	−0.0010	0.92	0.0087	0.10	−0.0021	0.85
DI	**−0.0543**	**6.6×10**^**−37**^	−0.0080	0.40	−0.0086	0.40	−0.0102	0.05	−0.0115	0.30
2hrGlu adj BMI	**0.0288**	**2.0×10**^**−13**^	0.0204	0.02	0.0064	0.49	**0.0292**	**2.26E**^**−9**^	−0.0257	0.01
FI	**−0.0033**	**4.4×10**^**−7**^	−0.0054	2.2×10^−4^	**0.0087**	**6.1×10**^**−8**^	**0.0068**	**1.96E**^**−16**^	**0.0071**	**3.8×10**^**−5**^
FI adj BMI	**−0.0026**	**4.4×10**^**−6**^	−0.0040	1.3 ×10^−3^	−0.0008	0.57	**0.0082**	**3.01E**^**−31**^	**0.0082**	**2.1×10**^**−8**^
HDL	−0.0008	0.51	−0.0031	0.27	−0.0059	0.05	**−0.0191**	**1.96E**^**−33**^	0.0069	0.038
Height	0.0009	0.12	**−0.0058**	**3.3×10**^**−5**^	**−0.0033**	**1.9×10**^**−5**^	**0.0061**	**4.44E**^**−05**^	−0.0005	0.77
HC	**−0.0031**	**3.5×10**^**−5**^	**−0.0113**	**1.0×10**^**−11**^	**0.0345**	**9.1×10**^**−79**^	**−0.0116**	**9.69E**^**−34**^	−0.0007	0.73
HOMA-B	**−0.0066**	**1.9×10**^**−21**^	**−0.0103**	**2.6×10**^**−11**^	**0.0066**	**8.0×10**^**−5**^	0.0019	0.03	0.0019	0.30
HOMA-IR	−0.0011	0.21	−0.0041	0.03	**0.0108**	**9.0×10**^**−8**^	**0.0066**	**2.2×10**^**−10**^	**0.0093**	**2.6×10**^**−5**^
Incr30	**−0.0398**	**6.9×10**^**−19**^	−0.0239	0.02	−0.0053	0.63	0.0198	4.8×10^−4^	0.0102	0.38
Ins30 adj BMI	**−0.0503**	**1.8×10**^**−28**^	−0.0310	1.8×10^−3^	0.0027	0.81	0.0163	3.9×10^−3^	0.0054	0.64
ISI adj BMI	−0.0039	0.06	−0.0020	0.67	0.0045	0.37	**−0.0213**	**1.3×10**^**−13**^	−0.0086	0.12
Leptin	0.0009	0.50	−0.0067	0.03	**0.0197**	**1.0×10**^**−9**^	**−0.0245**	**8.9×10**^**−21**^	**0.0147**	**3.1×10**^**−5**^
Linoleic acid	0.0093	0.29	−0.0232	0.25	0.0027	0.90	−0.0024	0.83	**0.1330**	**1.31x10^−8^**
Palmitoleic	0.0002	0.74	0.0024	0.11	0.0034	0.03	−0.0020	0.02	**−0.0104**	**5.50x10^−9^**
Proinsulin	**0.0097**	**1.2×10**^**−10**^	−0.0297	**1.4×10**^**−18**^	0.0047	0.18	0.0059	1.3×10^−3^	0.0059	0.13
Total Chol	0.0023	0.06	−0.0055	0.04	−0.0023	0.45	0.0046	3.2×10^−3^	**−0.0182**	**3.1×10**^**−8**^
Triglycerides	0.0022	0.07	−0.0027	0.33	0.0066	0.03	**0.0194**	**1.8×10**^**−34**^	**−0.0416**	**1.0×10**^**−35**^
Urate	−0.0007	0.51	−0.0045	0.084	**0.0165**	**1.4×10**^**−9**^	**0.0090**	**2.2×10**^**−10**^	**−0.0260**	**2.6×10**^**−18**^
WC	−0.0020	5.23×10^−3^	**−0.0096**	**1.5×10**^**−9**^	**0.0379**	**1.0×10**^**−102**^	**−0.0058**	**3.6×10**^**−10**^	−0.0005	0.80
WC female	−0.0010	0.30	−0.0073	3.9×10^−4^	**0.0376**	**1.4×10**^**−60**^	−0.0022	0.07	0.0000	0.99
WC male	−0.0031	1.6×10^−3^	**−0.0128**	**5.4×10**^**−9**^	**0.0374**	**1.1×10**^**−51**^	**−0.0102**	**2.8×10**^**−15**^	0.0007	0.80
WHR	0.0014	0.05	−0.0016	0.30	**0.0229**	**3.7×10**^**−39**^	**0.0051**	**1.6×10**^**−8**^	0.0016	0.43
WHR female	0.0027	4.0×10^−3^	0.0010	0.62	**0.0221**	**2.8×10**^**−22**^	**0.0140**	**5.6×10**^**−32**^	0.0027	0.31
WHR male	0.0003	0.74	−0.0049	0.03	**0.0242**	**1.4×10**^**−21**^	**−0.0059**	**7.6×10**^**−6**^	0.0003	0.92

Loci included in clusters

Beta Cell: *MTNR1B*, *CDKAL1*, *C2CD4A*, *HHEX*, *TCF7L2*, *SLC30A8*, *CDKN2A_B*, *CDC123*.*CAMK1D*, *HNF1A*, *AP3S2*, *ZHX3*, *UBE2E2*, *ACSL1*, *PRC1*, *GIPR HNF1B*, *KCNJ11*, *KCNQ1_2*, *ABO*, *ANK1*, *GLIS3*, *GLP2R*, *CTRB2*, *CDKN2A_2 DUSP8*, *ADCY5*, *GIP*, *HNF4A*, *HSD17B12 TLE4*.

Proinsulin: *ARAP1*, *SPRY2*, *DGKB_2*, *IGF2BP2*, *CCND2*, *HNF4A*, *CDC123*.*CAMK1D*.

Obesity: *FTO*, *MC4R*, *NRXN3*, *HSD17B12*, *RBMS1*.

Lipodystrophy: *IRS1*, *GRB14*, *PPARG*, *LYPLAL1*, *ANKRD55*, *CMIP*, *KLF14*, *LPL*, *ANKRD55_2*, *ARL15*, *ADCY5*, *C17orf58*, *POU5F1*, *MACF1*, *ZBED3*, *KIF9*, *ADAMTS9*, *CCND2*, *FAF1*, *MPHOSPH9*.

Liver: *GCKR*, *CILP2*, *HLA*.*DQA1*, *PNPLA3*, *TSPAN8*.*LGR5*.

*P*-values < 2 × 10^−4^ and corresponding betas are bolded, representing a Bonferonni correction of 47 traits × 5 clusters.

Abbreviations: BMI, body mass index; Chol, serum total cholesterol; CIR, corrected insulin response; DI, disposition index; FI, fasting insulin; FI adj BMI, FI adjusted for BMI; GWAS, genome-wide association study; HC, hip circumference; HDL, high-density lipoprotein; HOMA-B, homeostatic model assessment of beta cell function; HOMA-IR, homeostatic model assessment of insulin resistance; Incr30, incremental insulin response at 30 minutes on OGTT; Ins30 adj BMI, insulin response at 30 minutes on OGTT adjusted for BMI; ISI adj BMI, insulin sensitivity index adjusted for BMI; OGTT, oral glucose tolerance test; WC, waist circumference; WHR, waist-hip ratio; 2hrGlu adj BMI, 2-hour glucose on OGTT adjusted for BMI.

The most strongly weighted traits for Clusters 1 and 2 relate to insulin production and processing in the pancreatic beta cell. In Cluster 1 (Beta Cell cluster), these traits included decreased CIR, DI, insulin at 30 minutes of OGTT adjusted for BMI (Ins30 adj BMI), HOMA-B, and increased proinsulin levels adjusted for fasting insulin. Similarly, in Cluster 2 (Proinsulin cluster), the top-weighted traits included decreased Ins30 and HOMA-B but also decreased proinsulin levels adjusted for fasting insulin, suggesting another mechanism impacting beta cell function. The loci that clustered most strongly into Cluster 1 include many well-known beta cell–related loci: *MTNR1B*, *HHEX*, *TCF7L2*, *SLC30A8*, *HNF1A*, and *HNF1B* **(Table 1, S3 Table)** [60,61]. Similarly, the two strongest weighted loci in Cluster 2 are *ARAP1*, a locus at which diabetes risk is thought to be mediated by modulation of S*TARD10* expression in pancreatic beta cells [62], and *SPRY2*, a locus at which the closest gene, *SPRY2*, regulates insulin transcription [63] **(S3 Table)**. Using GWAS summary statistics, GRSs composed of risk alleles (“GWAS GRS”) from top-weighted loci in each cluster (*N* loci Beta Cell = 30, *N* loci Proinsulin = 7; see Materials and methods) were associated, as expected, with decreased HOMA-B (*P* < 10^−10^) in both clusters and fasting insulin (*P* < 5×10^−4^) in both clusters. The Beta Cell cluster GWAS GRS was associated with increased proinsulin (*P* < 10^−9^), while the Proinsulin cluster GWAS GRS was associated with decreased proinsulin (*P* < 10^−17^) (**Table 1**).

In contrast, Clusters 3, 4, and 5 all appeared to relate to mechanisms of insulin response. The traits that clustered most strongly with Cluster 3 (Obesity cluster) include increased WC, hip circumference (HC), BMI, and percent body fat. The top loci in this cluster include the well-known obesity-associated loci *FTO* and *MC4R* **(Table 1, S3 Table)**. The GWAS GRS for top-weighted loci in this cluster (*N* loci = 5) was significantly associated with these same traits (*P* < 10^−24^) as well as increased fasting insulin unadjusted for BMI (*P* < 10^−7^), but not fasting insulin adjusted for BMI (*P* = 0.57). Thus, based on these association patterns, this cluster appeared to represent an obesity-mediated form of insulin resistance.

Cluster 4 (Lipodystrophy cluster) appears to represent the same “lipodystrophy-like” insulin resistance cluster previously suggested by Yaghootkar and colleagues [10,64], with all the variants from that previous set that were also associated with T2D being among those loci most strongly weighted in this cluster (*PPARG*, *ANKRD55*, *ARL15*, *GRB14*, *IRS1*, and *LYPLAL1*) **(Table 1, S3 Table)**. Cluster 4’s strongest weighted traits were decreased ISI adjusted for BMI, adiponectin, HDL, and increased triglycerides; GWAS GRSs of alleles from the strongest weighted loci in this cluster (*N* loci = 20) were associated with all of these traits (*P* < 10^−20^). This cluster appears to represent a lipodystrophy or fat distribution–mediated form of insulin resistance. Interestingly, while an increased GWAS GRS from this cluster was significantly associated with increased WHR in women (*P* < 10^−31^), it was associated with decreased WHR in men (*P* < 10^−5^). These ratios appear to be driven by GWAS GRS associations with decreased HC in both sexes (*P* < 10^−9^) but a significant association with decreased WC only observed in men (*P* < 10^−14^).

Cluster 5 (Liver/Lipid cluster) was notable for having decreased serum triglyceride levels, palmitoleic acid, urate, and linolenic acid, along with increased gamma-linolenic acid, as the traits most strongly weighted in this cluster. The GWAS GRS for the highest weighted loci (*N* loci = 5) in this cluster was significantly associated with all the above traits (*P* < 10^−7^). Notably, three of the top four weighted loci, *GCKR*, *CILP2/TM6SF2*, and *PNPLA3* **(Table 1, S3 Table)** have been previously associated with nonalcoholic fatty liver disease (NAFLD) [65], and functional work has implicated these loci in liver lipid metabolism [66–70].

### Clusters are distinctly enriched for tissue enhancers or promoters

**Fig 2:**
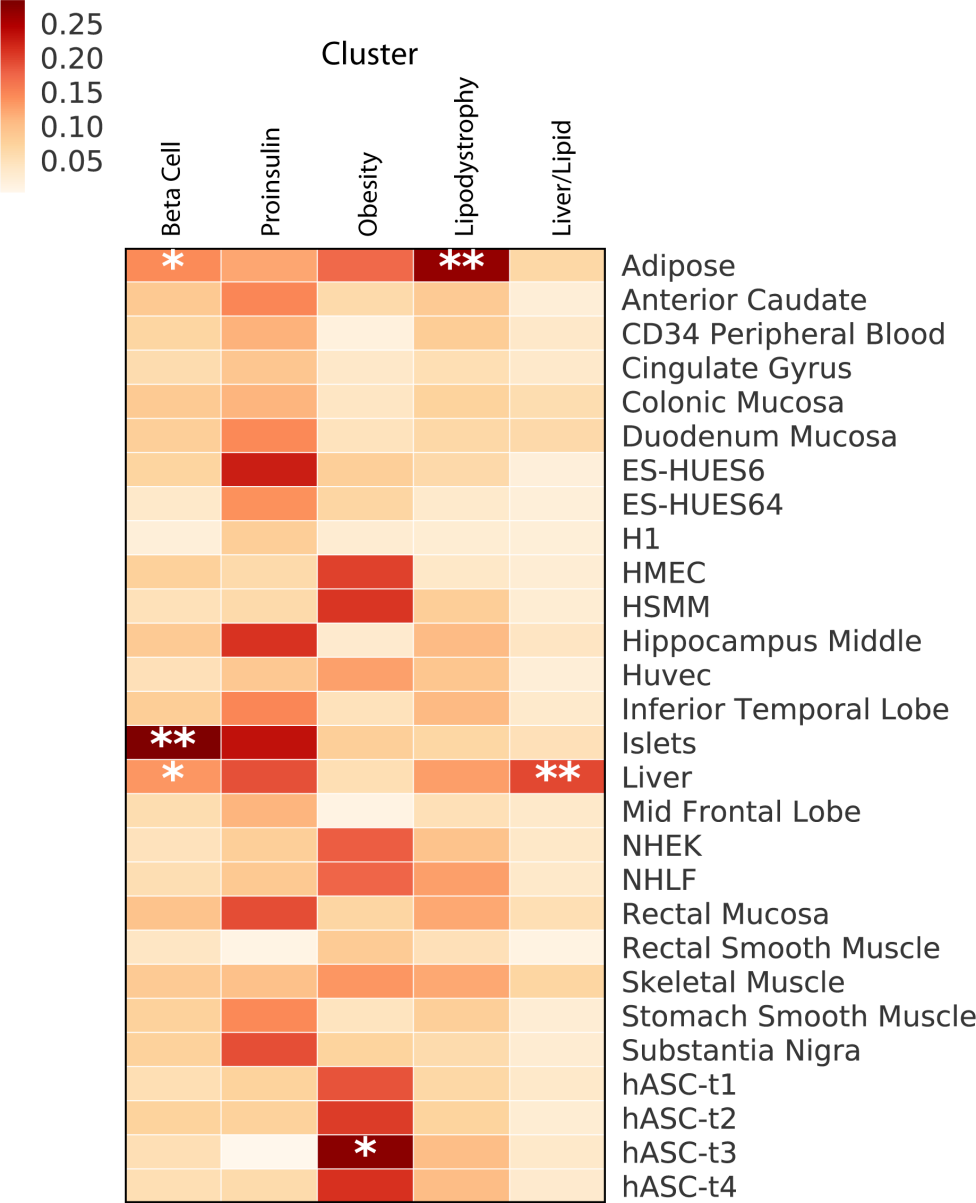
Enrichment for tissue-specific enhancers in clusters. Heatmap of associations of enrichment for enhancers and promoters from tissues residing within the top-weighted loci from each cluster. **P < 0.001, *P < 0.05. Cell line epigenetic data were obtained through the Epigenomics Roadmap; the authors played no role in the procurement of tissue or generation of the data for the three embryonic stem cell lines. CD34, Cluster of differentiation 34; ES-HUES6, HUES6 human embryonic stem cell line; ES-HUES64, HUES64 human embryonic stem cell line; hASC, human Adipose-derived Stromal Cells; HMEC, Human Mammary Epithelial Cell line; HSMM, Human Skeletal Muscle Myoblast cell line; H1, H1 human embryonic stem cell line; NHEK, Normal Human Epidermal Keratinocyte cell line; NHLF, Normal Human Lung Fibroblast cell line.

To gain further support for the suspected mechanistic pathways represented by these clusters and assess the biological distinctness of the clusters through an independent analysis, we assessed the top loci in each cluster for enrichment of epigenomic annotations **(Fig 2)**. We expected each pathway to capture a different disease mechanism and thus localize largely to specific and distinct tissues; as common variants have been shown to lie typically in noncoding regions and presumably alter regulatory elements (enhancers, promoters), we assessed whether variants in the credible sets for loci in each cluster preferentially altered enhancers or promoters in specific cell types.

Within the Liver/Lipid cluster, there was significant enhancer/promoter enrichment in liver tissue (*P* < 0.001), and within the Lipodystrophy cluster, there was significant enrichment in adipose tissue (*P* < 0.001), each compared to the 27 other tissues assessed **(Fig 2)**. The adipose tissue enrichment in the Lipodystrophy cluster was also the most significant across the five clusters (*P* < 0.05) (**S5 Fig**). The loci in the Obesity cluster were most strongly enriched for enhancers/promoters in pre-adipose tissue, both compared to the other tissues and clusters (*P* < 0.05). The two clusters suspected to be involved in beta cell function (Beta Cell cluster and Proinsulin cluster) were most strongly enriched for pancreatic islet cell enhancers and promoters, with significant within (*P* < 0.001) and across (*P* < 0.05) enrichment for the Beta Cell cluster. Interestingly, the Proinsulin cluster had distinct enrichment in embryonic stem cells and brain tissue compared to the other clusters (*P* < 0.05), which may reflect gene regulation related to early development, as beta cells and neurons have been thought to share gene expression patterns related to development [71]. Thus, in each case, the tissue enrichment supported the predicted biological mechanisms of each cluster.

### Clusters are differentially associated with clinical outcomes from GWAS

To establish translational relevance, we next asked whether GWAS GRSs from the strongest weighted loci in each cluster were associated with any clinical outcomes. We focused on clinical outcomes related to T2D available through GWAS: CAD, renal function as assessed by eGFR and UACR, stroke risks, and blood pressure measures (**Table 2**). GWAS GRSs from the Beta Cell and Lipodystrophy clusters were most strongly associated with increased risk of CAD (*P* < 10^−7^). Increased Beta Cell cluster GWAS GRS was also significantly associated with increased risk for ischemic stroke (*P* = 10^−4^) as well as stroke subtypes, including large artery (*P* = 10^−5^) and small vessel disease–related strokes (*P* = 10^−4^), but not cardioembolic stroke (*P* = 0.6). There were also nominally significant trends for these same stroke subtypes with the Lipodystrophy cluster. Only increased Lipodystrophy cluster GWAS GRS was significantly associated with increased blood pressure (SBP, *P* = 6 × 10^−6^; DBP, *P* = 5 × 10^−9^). Renal function was most significantly associated with the Liver/Lipid cluster GWAS GRS; interestingly, there was a significant association for increased Liver/Lipid GWAS GRS with reduced eGFR (*P* = 10^−6^) but, surprisingly, also reduced UACR (*P* = 10^−3^), whereas typically patients with diabetic kidney disease have reduced eGFR with elevated UACR. In contrast, increased Lipodystrophy cluster GWAS GRS was significantly associated with increased UACR (*P* = 9 × 10^−5^). No cluster GWAS GRS was associated with risk of CKD, defined as eGFR < 60 mL/minute/1.73 m^2^.

**Table 2:** Associations of cluster genetic risk scores and clinical outcomes from GWAS.

	Beta Cell *N* Loci = 30		Proinsulin *N* Loci = 7		Obesity *N* Loci = 5		Lipodystrophy *N* Loci = 20		Liver/Lipid *N* Loci = 5		Loci Combined *N* Loci = 62	
Outcome	beta	*P*-value	beta	*P*-value	beta	*P*-value	beta	*P*-value	beta	*P*-value	Beta	*P*-value
CAD	**0.021**	**2.08E**^**−12**^	−0.003	0.67	0.016	0.04	**0.021**	**2.5×10**^**−8**^	−0.009	0.27	0.017	**1.2×10**^**−15**^
CKD	0.003	0.35	0.009	0.22	0.015	0.04	0.0002	0.97	0.011	0.18	0.004	0.06
eGFR	0.000	0.87	0.0002	0.70	−0.0008	0.06	−0.00003	0.89	**−0.002**	**1.2×10**^**−6**^	0.000	0.07
UACR	0.001	0.42	0.003	0.27	0.003	0.32	**0.006**	**9.0×10**^**−5**^	−0.010	3.7×10^−3^	0.002	0.01
Stroke_IS	**0.016**	**2.0×10**^**−4**^	0.009	0.37	0.022	0.03	0.014	9.1×10^−3^	−0.002	0.84	**0.013**	**1.3×10**^**−5**^
Stroke_CE	0.004	0.59	−0.001	0.94	0.048	0.01	0.005	0.62	0.002	0.93	0.007	0.20
Stroke_LVD	**0.032**	**5.6×10**^**−5**^	−0.006	0.73	0.020	0.31	0.026	0.01	−0.017	0.46	**0.023**	**5.1×10**^**−5**^
Stroke_SVD	**0.032**	**2.6×10**^**−4**^	0.029	0.17	0.027	0.22	0.036	1.5×10^−3^	−0.007	0.79	**0.028**	**7.3×10**^**−6**^
SBP	0.035	0.09	−0.014	0.76	−0.088	0.07	**0.149**	**4.9×10**^**−9**^	−0.041	0.45	0.048	9.3×10^−4^
DBP	0.014	0.30	−0.027	0.36	−0.046	0.14	**0.073**	**6.4×10**^**−6**^	0.0005	0.99	0.023	0.01

*P*-values < 8 × 10^−4^ and corresponding betas are bolded, representing a Bonferonni correction of 10 outcomes × 6 groups.

Abbreviations: CAD, coronary artery disease; CE, cerebroembolic; CKD, chronic kidney disease; DBP, diastolic blood pressure; eGFR, estimated glomerular filtration rate; IS, ischemic stroke all subtypes; LVD, large vessel disease; SBP, systolic blood pressure; SVD, small vessel disease; UACR, urine albumin-creatinine ratio.

### Application of clusters to patients with T2D

To determine whether cluster GRS associations observed in large GWAS would have relevance to patients with T2D, we investigated cohorts of individuals with T2D ascertained from two epidemiological studies (METSIM [*N* = 487] and the Diabetes Genes in Founder Populations [Ashkenazi] Study [*N* = 509]) and two from biobanks (the Partners Biobank [*N* = 2,065] and the UKBB [*N* = 14,813]).

We first validated associations between cluster GRSs and predicted phenotypes. In the up to 17,365 combined individuals with T2D from the four cohorts, increased individual-level GRSs were associated with the expected salient traits (**Fig 1B, S6 Table**): decreased BMI and percent body fat (*P* < 10^−20^) as well as fasting C-peptide (*P* = 5 × 10^−5^) in the Beta Cell cluster; similarly decreased BMI (*P* = 3 × 10^−8^) and fasting C-peptide (*P* = 0.04) in the Proinsulin cluster; increased BMI, percent body fat, HC, and WC (*P* < 10^−5^) in the Obesity cluster; decreased BMI, percent body fat (*P* < 10^−15^), and HDL (*P* = 5 × 10^−5^) in the Lipodystrophy cluster; and decreased triglycerides in the Liver/Lipid cluster (*P* = 2 × 10^−5^). Interestingly, as was noted with the GWAS GRS, a higher individual-level Lipodystrophy GRS had sex-differential associations with anthropometric traits: increased WHR in women (*P* = 0.007) but decreased in men (*P* = 0.005). A similar discrepant pattern was also seen with BMI adjustment (**S6 Table**).

We next asked whether this genetic approach could be used to identify individuals with T2D who had cluster-specific characteristics, in an initial attempt to stratify the population. In other words, would individuals with the largest GRSs for each cluster differ from each other and all other individuals with T2D, with regard to any clinical traits?

Consistently across the studies (*N* T2D = 17,365), we observed about 30% of individuals with a GRS at the top 10th percentile of just one cluster (**S8 Table**), which is what is expected by chance (under a binomial distribution). These individuals with the highest GRSs differed significantly from all other individuals with T2D (**Fig 1C, S7,** and **S9 Tables**); for instance, compared to all individuals across the three studies with T2D, those with extreme GRS in the Beta Cell cluster (*N* = 1,068) had decreased BMI, HC, and WC (*P* < 10^−3^) and percent body fat (*P* < 0.05), with a trend toward decreased fasting C-peptide (*P* = 0.19), and those in the Proinsulin cluster (*N* = 1,117) had significantly decreased fasting C-peptide levels (*P* = 0.003). Those in the Obesity cluster (*N* = 1,206) had increased BMI, percent body fat, HC, and WC (*P* < 0.05); those with extreme GRS in the Lipodystrophy cluster (*N* = 1,134) had significantly decreased HDL, percent body fat, and BMI (*P* < 0.01), and those with extreme GRS in the Liver/Lipid cluster (*N* = 924) had significantly decreased triglycerides (*P* = 0.01). Thus, individuals with T2D and a GRS uniquely at the top 10th of one cluster as a group had representative trait characteristics distinguishing them from all other individuals with T2D (**Fig 1C, S7,** and **S9 Tables**).

## Discussion

T2D, typically defined as hyperglycemia that is not autoimmune or monogenic in origin, is commonly recognized as a heterogeneous conglomerate of various pathogenic mechanisms and therefore is unlikely to represent a single disease process. However, understanding of the biological pathways causing T2D to inform clinical management remains incomplete. Furthermore, despite over 100 T2D loci now identified, the relationship of these loci to disease pathways remains largely opaque.

The work described here is the most comprehensive assessment of T2D loci clustering to date, including variant-trait associations for 94 T2D genetic variants and 47 diabetes-related metabolic traits in publicly available GWAS datasets. We identify five robust clusters of T2D variants, which appear to represent biologically meaningful distinct pathways. The first two clusters (Beta Cell and Proinsulin) relate to pancreatic beta cell function and differ most notably in the direction of association with proinsulin; both contain loci for which functional work has implicated beta cell function in the causal mechanisms (e.g., [60,61]). Additionally, the loci in both these clusters were highly enriched for positional overlap with pancreatic islet tissue enhancers and promoters. The three other clusters (Obesity, Lipodystrophy, Liver/Lipid) appear to represent different pathways causing insulin resistance: obesity mediated, lipodystrophy (fat-distribution) mediated, and liver-lipid-metabolism mediated. The Liver/Lipid cluster contains three of the top loci associated with NAFLD [65], and functional work has implicated these loci in liver lipid metabolism resulting in sequestration of lipid in the liver, resulting in decreased observed serum triglyceride levels [66–70]. Additionally, these three clusters related to insulin action (Obesity, Lipodystrophy, Liver/Lipid) are enriched for variants overlaying enhancers in tissues that biologically support their proposed mechanisms: pre-adipocytes, adipocytes, and liver tissue, respectively. GRSs of top-weighted loci from the five clusters were also associated with particular clinical outcomes, including increased SBP, risk of CAD, and stroke, assessed using GWAS summary statistics.

Previous clustering efforts of T2D loci included less than half as many diabetes-related traits [6,9,11] and focused predominantly on unsupervised hierarchical clustering, a method that involves “hard clustering,” whereby a locus can be a member of only one cluster. Our analysis uses a novel clustering method, bNMF, which enables "soft clustering," whereby a variant can be a member of more than one cluster and also a data-driven method for determining the number of clusters. With this method, the derived clusters are biologically interpretable and include mechanistic processes not previously captured by previous hard-clustering efforts, such as the Liver/Lipid cluster.

Our study additionally asked whether the clusters of variants have relevance to individuals with T2D. In four cohorts with up to 17,365 individuals with T2D of European ancestry, we showed that individual-level GRSs for each cluster were associated significantly with predicted traits. Additionally, individuals with a very high GRS uniquely in the top 10th percentile of one cluster had clinical features significantly distinguishing themselves from all other individuals with T2D; we observed consistently that this group comprised about 30% of persons with T2D, consistent with chance expectation and, importantly, representing a sizable proportion of individuals with T2D.

Thus, these results suggest that genetics can be used to stratify a reasonable proportion of individuals with T2D who may belong to clinically meaningful subgroups. Such individuals could be classified based on their genetics and targeted for precision surveillance and therapeutics, should future studies find that these individuals differentially respond to medical interventions or confirm risk of particular clinical outcomes. Of course, the threshold we chose of the top 10th percentile for each cluster GRS is arbitrary, and further work is needed to determine clinically relevant thresholds or combinations of GRS from multiple clusters. Using the 10th percentile cutoff, study individuals with extreme GRSs on aggregate had clinical characteristics distinguishable from others with T2D; however, at the individual level, such clinical features might not be recognizable to a clinician because of the subtleties of the phenotypic characteristics, small differences in effects sizes, and/or the potential for environmental influences. Furthermore, as germline genetic variation is constant throughout the lifetime and essentially unaltered by medications, it may provide a more robust metric than other biomarkers (which are contingent on environmental changes), on which to anchor an initial stratification step.

Other efforts have tried to identify subtypes of T2D [3,4]. In the most recent assessment of patients with new adult-onset diabetes in Scandinavia, Ahlqvist and colleagues used phenotypic information to define five subgroups of diabetes: an autoimmune form (capturing type 1 diabetes and latent autoimmune diabetes in the adult), two severe forms (severe insulin-deficient [SIDD] and severe insulin-resistant diabetes [SIRD]), and two mild forms (obesity- and age-related diabetes) [4]. Importantly, in contrast to our clusters of genetic loci, these clusters are defined using clinical data and biomarkers at the time of diabetes diagnosis, and thus analysis of the same variables at a different time in the disease course or after treatment could yield inappropriate results. The T2D subtypes described by Ahlqvist and colleagues seemed to have different genetic architectures [4], so we were interested to determine whether our clusters of genetic loci corresponded. Of the ten variants Ahlqvist and colleagues found to be associated at nominal significance with their SIDD cluster that were also included in our analysis, seven of these variants (or a proxy) had their strongest weights in our Proinsulin or Beta Cell clusters. Our Obesity cluster may correspond to the mild obesity-related diabetes of Ahlqvist and colleagues; however, none of the top-weighted variants in this cluster were included in their analysis. To the extent that severity of disease might be correlated with duration of exposure (with genetic exposure present at conception), our insulin resistance–related clusters might correspond to the SIRD of Ahlqvist and colleagues; there were four variants found by Ahlqvist and colleagues to be associated with the SIRD cluster at nominal significance, all of which were included in our analysis and had their strongest weights in clusters we believe to relate to insulin resistance (Liver/Lipid and Lipodystrophy clusters). Interestingly, variants from several of our clusters were associated with the age-related diabetes cluster from Ahlqvist and colleagues.

Beyond clarifying disease causal mechanisms and offering the potential for patient stratification, identification of the biologically motivated clusters of T2D loci in our study may help also implicate loci with unknown function into pathways. For example, it is interesting that the *HLA*.*DQA1* locus is most highly weighted in the Liver/Lipid cluster; one might expect it to have a predominant autoimmune mechanism of action, given its chromosomal location in the human leukocyte antigen (HLA) region. The function of this locus remains to be discovered; however, our results suggest that it is more likely to influence insulin resistance than insulin deficiency. Membership to clusters may thus facilitate characterization of loci, which are generally challenging to study functionally.

Five variants were top weighted (above the threshold of 0.75) in more than one cluster: *CDC123/CAMKID* (Beta Cell and Proinsulin), *HSD17B12* (Beta Cell and Obesity), *ADCY5* (Beta Cell and Lipodystrophy), *CCND2* (Proinsulin and Lipodystrophy), and *HNF4A* (Beta Cell and Proinsulin). A strong weighting in more than one cluster suggests that these loci are involved in more than one mechanistic process. Published functional data support such pleiotropy; for example, the gene product of *CCND2* is thought to be involved in pancreatic beta cell growth [72] (in line with the Proinsulin cluster) and also in early stage adipocyte differentiation [73] (consistent with the Lipodystrophy cluster).

Several loci included in this analysis have evidence of multiple independent signals [6,8]. We included 15 variants from 6 such loci (*ANKRD55*—two variants, *DGKB*—two variants, *CDKN2A*—three variants, *KCNQ1*—four variants, *CCND2*—two variants, and *HNF4A*—two variants), offering an opportunity to see whether distinct signals from the same locus would cluster together (**S1 Table**). The multiple variants in *ANKRD55*, *CDKN2A*, *KCNQ1*, and *CCND2* mapped most strongly to the same cluster for each locus. At the *DGKB* locus, the signal represented by rs10276674 was most strongly weighted in the Proinsulin cluster, whereas the signal represented by rs2191349 was strongly weighted in the Beta Cell and Liver/Lipids clusters. In *HNF4A*, rs4812829 was most strongly weighted in the Beta Cell and Proinsulin clusters, whereas rs1800961 was most strongly weighted in the Lipodystrophy, Proinsulin, and Liver/Lipid clusters (**S3 Table**). Interestingly, therefore, for *HNF4A*, the two signals separate into predominant insulin deficiency and insulin resistance–related mechanisms. Potentially, therefore, cross-phenotype analysis can provide additional support for the existence of independent signals at these loci, perhaps indicating tissue-specific regulatory mechanisms.

The strengths of this study include the novel application of a Bayesian form of NMF clustering to complex disease genetics. The clusters resulting from bNMF are more readily interpretable than hierarchical clustering, given that bNMF also clusters traits. Furthermore, allowance of an element to be part of more than one cluster (soft clustering) fits with our biological understanding of complex disease-causing genetic variants, whereby a particular variant may impact more than one biological pathway.

Limitations of this study include clustering of only available phenotypes from GWAS. It is possible that future inclusion of additional traits would impact the clustering results, potentially even creating a new cluster for a mechanistic pathway not currently captured with available phenotypes. Our analysis considered all known published T2D loci reaching genome-wide significance to date; we expect that with forthcoming larger GWAS, additional loci will be identified and could be included in future analyses [74]. Additionally, we have focused on variants associated with T2D and related traits in populations of European ancestry. With additional studies from populations of diverse ethnic backgrounds, it would be ideal to include additional T2D-associated variants that were not included in the current analysis. Furthermore, the impact of the clusters on outcomes such as stroke and CAD was assessed through GWAS GRS using GWAS summary statistics, which came from studies including individuals with and without diabetes. It will be important to assess the association of cluster GRS and these outcomes in future work using individual-level data; such analyses would ideally involve cohorts with large sample sizes and well-phenotyped outcomes. While NMF provides a very attractive method for variant-trait clustering, it is currently uncertain whether all weights or a thresholded approach is ideal for assignment of elements in a cluster. For our analysis, we developed a framework to determine a reasonable threshold; however, this question could benefit from additional research.

In summary, clustering of genetic variants associated with T2D has identified five robust clusters with distinct trait associations, which likely represent mechanistic pathways causing T2D. These clusters have distinct tissue specificity, and patients enriched for alleles in each cluster exhibit distinct predicted phenotypic features. We observe a substantial fraction (about 30%) of individuals with T2D who have T2D genetic risk factors highly loaded (in top 10th percentile) from just one of the five clusters. It will be exciting to explore whether such individuals respond differentially to medications based on the pathway predominantly disrupted or whether they have a differential rate of disease progression and diabetic complications. Classification of patients using data from designated genetic pathways may offer a step toward genetically informed patient management of T2D in order to individualize and improve care.

## Supporting information

S1 Fig Loci associations to clusters. (TIF)

S2 Fig Trait associations to clusters. (TIF)

S3 Fig Distribution of cluster weights for variants. (TIF)

S4 Fig Deltas of cluster weights. (TIF)

S5 Fig Enrichment for tissue-specific enhancers and promoters compared across clusters. (TIF)

S1 Table List of SNPs. (XLSX)

S2 Table GWAS datasets. GWAS, genome-wide association study.
(XLSX)

S3 Table Clustering results for T2D loci. T2D, type 2 diabetes.
(XLSX)

S4 Table Clustering results for traits. (XLSX)

S5 Table GWAS GRS associations with traits. GRS, genetic risk score; GWAS, genome-wide association study.
(XLSX)

S6 Table Meta-analysis of GRS associations with traits in four studies of patients with T2D (adjusted for age and sex) plus sex-specific analyses. GRS, genetic risk score; T2D, type 2 diabetes.
(XLSX)

S7 Table Meta-analysis of comparison of mean trait in individuals with top 10% decile of one cluster GRS (cluster extreme) versus all subjects with T2D in four studies. GRS, genetic risk score; T2D, type 2 diabetes.
(XLSX)

S8 Table Counts of individuals from five studies in uniquely one top 10% decile of cluster GRSs ("cluster extreme"). GRS, genetic risk score.
(XLSX)

S9 Table Comparison of median values of traits in "cluster extreme" (top decile GRS) groups and in all others with T2D in two biobanks. GRS, genetic risk score; T2D, type 2 diabetes.
(XLSX)

## Abbreviations

BMI: body mass index
bNMF: Bayesian nonnegative matrix factorization
CAD: coronary artery disease
CIR: corrected insulin response
CKD: chronic kidney disease
DBP: diastolic blood pressure
DI: disposition index
DIAGRAMv3: DIAGRAM version 3
eGFR: estimated glomerular filtration rate
EGG: Early Growth Genetics
EMR: electronic medical record
FI adj BMI: FI adjusted for BMI
GIANT: Genetic Investigation of ANthrometric Traits
GRS: genetic risk score
GWAS: genome-wide association study
HbA1c: glycated hemoglobin
HC: hip circumference
HDL: high-density lipoprotein
HLA: human leukocyte antigen
HOMA-B: homeostatic model assessment of beta cell function
HOMA-IR: homeostatic model assessment of insulin resistance
HRC: Haplotype Reference Consortium
Incr30: incremental insulin response at 30 minutes of OGTT
Ins30: insulin at 30 minutes of OGTT
Ins30 adj BMI: Ins30 adjusted for BMI
ISI: insulin sensitivity index
LDL: low-density lipoprotein
MAGIC: Meta-Analyses of Glucose and Insulin-related traits Consortium
METSIM: Metabolic Syndrome in Men Study
NAFLD: nonalcoholic fatty liver disease
NMF: nonnegative matrix factorization
OGTT: oral glucose tolerance test
RPDR: Research Patient Data Registry
SBP: systolic blood pressure
SIDD: severe insulin-deficient diabetes
SIRD: severe insulin-resistant diabetes
T2D: type 2 diabetes; UACR, urine albumin-creatinine ratio
UKBB: UK Biobank
WC: waist circumference
WHR: waist-hip ratio
2hrGlu adj BMI: 2-hour glucose on oral glucose tolerance test adjusted for body mass index
