# Supplementary figures and images for: Type 2 diabetes genetic loci informed by multi-trait associations point to disease mechanisms and subtypes: A soft clustering analysis
Source: PLoS Med. 2018 Sep 21;15(9):e1002654. doi: 10.1371/journal.pmed.1002654 (PMC6150463; doi:10.1371/journal.pmed.1002654)

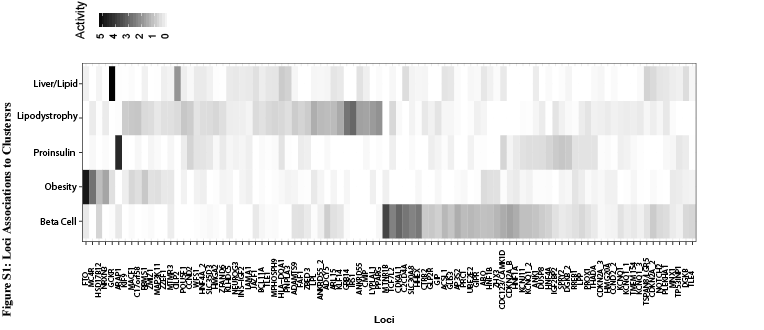

Supplement: S1 Fig — (TIF) [file pmed.1002654.s001.tif]

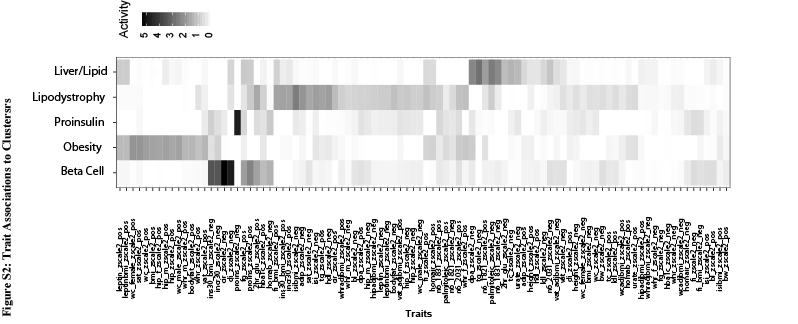

Supplement: S2 Fig — (TIF) [file pmed.1002654.s002.tif]

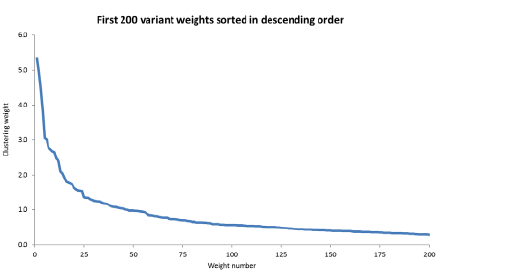

Supplement: S3 Fig — (TIF) [file pmed.1002654.s003.tif]

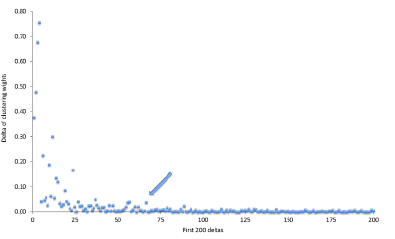

Supplement: S4 Fig — (TIF) [file pmed.1002654.s004.tif]

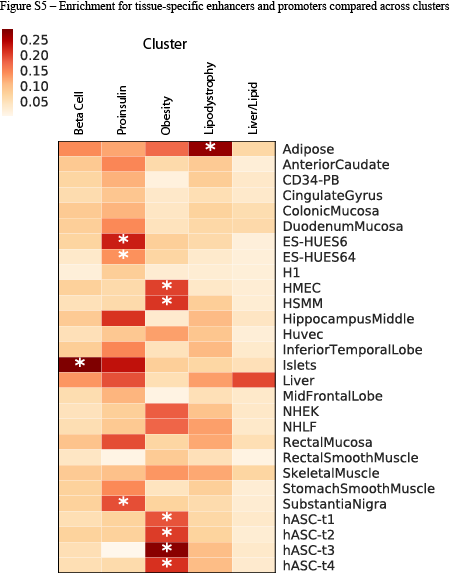

Supplement: S5 Fig — (TIF) [file pmed.1002654.s005.tif]
